# Influence of Pre-Existing Pain on the Body’s Response to External Pain Stimuli: Experimental Study

**DOI:** 10.2196/70938

**Published:** 2025-08-20

**Authors:** Burcu Ozek, Zhenyuan Lu, Srinivasan Radhakrishnan, Sagar Kamarthi

**Affiliations:** 1 Mechanical and Industrial Engineering Department, Northeastern University, 360 Huntington Avenue, Boston, MA, 02115, United States, 1 6173733070

**Keywords:** pain measurement, sensors, physiological signals, hypothesis testing, pain assessment, ANOVA

## Abstract

**Background:**

Accurately assessing pain severity is essential for effective pain treatment and desirable patient outcomes. In clinical settings, pain intensity assessment relies on self-reporting methods, which are subjective to individuals and impractical for noncommunicative or critically ill patients. Previous studies have attempted to measure pain objectively using physiological responses to an external pain stimulus, assuming that the participant is free of internal body pain. However, this approach does not reflect the situation in a clinical setting, where a patient subjected to an external pain stimulus may already be experiencing internal body pain.

**Objective:**

This study investigates the hypothesis that an individual’s physiological response to external pain varies in the presence of preexisting pain.

**Methods:**

We recruited 39 healthy participants aged 22‐37 years, including 23 female and 16 male participants. Physiological signals, electrodermal activity, and electromyography were recorded while participants were subject to a combination of preexisting heat pain and cold pain stimuli. Feature engineering methods were applied to extract time-series features, and statistical analysis using ANOVA was conducted to assess significance.

**Results:**

We found that the preexisting pain influences the body’s physiological responses to an external pain stimulus. Several features—particularly those related to temporal statistics, successive differences, and distributions—showed statistically significant variation across varying preexisting pain conditions, with *P* values <.05 depending on the feature and stimulus.

**Conclusions:**

Our findings suggest that preexisting pain alters the body’s physiological response to new pain stimuli, highlighting the importance of considering pain history in objective pain assessment models.

## Introduction

Accurate pain assessment is vital for ensuring proper treatment and helping patients receive the necessary care to reduce discomfort and prevent complications. Yet, current pain assessment tools and methods, which rely on patients’ description of their pain using scales or descriptive measures, often fall short of clinical expectations [1]. These methods are ineffective for noncommunicative patients, such as infants or critically ill patients under sedation or mechanical ventilation. They are also inherently subjective, as pain perception varies widely between individuals [2-5]. These limitations increase the risk of misdiagnosis and mistreatment, highlighting the need for more objective and reliable pain assessment methods [6,7].

To address the limitations of self-reported pain assessments, physiological signals offer a promising alternative. Signals such as skin conductance, heart rate, and muscle activity provide objective data that can reflect the body’s response to pain. Unlike self-reporting, physiological signals do not depend on a patient’s ability to communicate, making them particularly suitable for critically ill or noncommunicative patients. By monitoring these signals in real-time, health care providers can gain an accurate and continuous understanding of a patient’s pain levels, paving the way for timely and appropriate interventions. This shift toward objective, data-driven pain assessment can help reduce the variability and inaccuracies associated with traditional methods, enhancing health care providers’ assessments [8,9].

Several studies have explored data-driven approaches for assessing pain through physiological signals [10-12]. These studies primarily collected data such as skin conductance, electromyography (EMG), electrocardiography, and electroencephalography during controlled pain stimuli experiments [9,13-15]. The BioVid Heat Pain Database is one of the most well-known, aiming to differentiate between various pain levels by analyzing physiological responses to heat pain [9]. Other studies, like Rojas et al [16] and Lin et al [14], also gathered data from participants exposed to heat or cold stimuli, applying machine learning techniques to classify pain levels. These studies have demonstrated the potential of physiological signals for objective pain assessment and established valuable datasets for pain assessment research [9,14,17,18].

While the aforementioned studies provide promising results, they mainly focus on healthy participants responding to a single type of externally induced pain stimulus. One crucial factor that remains underexplored is the impact of preexisting conditions, such as chronic pain, postsurgical pain, or injury pain, that a patient is experiencing when the patient is administered an external pain stimulus. A few studies have investigated different patient populations, such as patients with chronic pain (back pain and shoulder pain) [11,19-22], patients in postsurgery [23], patients who are injured [24], patients with orthopedic trauma [25], patients with musculoskeletal trauma [26], and patients with cancer (eg, breast cancer [27]). These studies have provided insights into pain assessment in these populations, but they have not fully explored how preexisting pain interacts with new pain stimuli in terms of physiological responses.

Although the literature has begun exploring objective pain assessment for a single source of external pain stimuli, insights from medical research reveal that preexisting pain influences responses to new pain stimuli, underscoring the importance of considering preexisting pain. Sacco et al [20] found that individuals without chronic pain (without preexisting pain) exhibit an adaptive response to acute pain (new pain) by activating internal pain regulation mechanisms, including the release of natural painkillers and an increase in blood pressure, which temporarily reduces sensitivity. However, in patients with chronic pain, this adaptive mechanism can become disrupted, leading to heightened sensitivity to both acute and chronic pain. Similarly, Moscato et al [22] found that the autonomic signals of patients with chronic low back pain show differences compared to those of healthy individuals, both at rest and when subjected to a noxious stimulus, as evaluated through a set of physiological indicators. Lee et al [26] showed that preexisting pain can impact specific biomarkers, such as IL−1β, affecting how the body processes musculoskeletal trauma as a new pain. Raza et al [27] also found that women with chronic breast pain experienced more severe postoperative pain, highlighting preexisting pain as a predictor of adverse pain outcomes. In patients with trauma, Fetzh et al [24] observed that preexisting pain serves as a significant predictor for long-term pain following severe injury, emphasizing the complex interaction between pain history and physiological responses.

Although chronic pain is often referenced in the literature, the goal of this study is neither to simulate nor to assess chronic pain specifically. Instead, we use “preexisting pain” as a broader effect that can include various types of ongoing pain, such as postsurgical pain, injury-related pain, or other chronic and nonchronic conditions. Our aim is to investigate how any form of preexisting pain—regardless of origin—might influence the physiological response to a new external pain stimulus.

Our hypothesis is that preexisting pain significantly alters physiological responses to new pain stimuli. For instance, patients with chronic pain or postsurgical pain may show distinct physiological signals—such as changes in skin conductance or EMG—compared to healthy individuals when encountering new pain. To test this hypothesis, we conducted an experimental study examining how different levels of preexisting pain influence physiological responses to new pain stimuli. Understanding these responses could lead to accurate and personalized pain assessments.

In our experiments, we designated “heat pain” as a form of preexisting pain and “cold pain” as a new external stimulus. Heat pain and cold pain were studied at 3 levels: zero, low, and high. We conducted experiments with 9 combinations of no-heat, low-heat, high-heat, no-cold, low-cold, and high-cold pain. We recorded electrodermal activity (EDA) and EMG as time series data during these experimental conditions. Following data collection, we used feature engineering methods to extract features from these time series. We identified distributions, simple temporal statistics, linear and nonlinear autocorrelation, successive differences, and fluctuation analysis as pain-sensitive features. Next, we applied an ANOVA test to investigate whether physiological responses to cold pain stimuli exhibit statistical differences across three levels of preexisting heat pain. By analyzing variations in EDA and EMG features across different pain exposure levels, we aim to gain insights into how preexisting pain modulates the body’s response to new pain.

The aim of this study is to investigate how varying levels of preexisting heat pain affect the physiological response to new cold pain stimuli, using EDA and EMG signals as objective markers.

To our knowledge, this work represents the first experimental study that explores the EDA and EMG features that exhibit statistically significant differences across varying preexisting heat pain levels in response to an external stimulus.

## Methods

### Ethical Considerations

The research protocol was approved by the Northeastern University Institutional Review Board (IRB #22-11-06). The methods for this study adhered to the guidelines outlined in the Belmont Report. Northeastern University holds a Federal Wide Assurance with the US Department of Health & Human Services, ensuring our compliance with the principles of the Common Rule, 45 CFR 46. Before the experiment, the researcher orally explained the experimental procedure to each participant, the participant’s role, and other relevant information. In addition, the researcher presented each participant with a written consent form to read. The researcher obtained written informed consent from each participant before commencing the experiment. The research team kept participants’ data confidential and anonymized, securely storing all data with access limited to the research team only. No identifying information was included in the manuscript or any related materials. Participants were compensated with a gift card.

### Participants

In total, 39 participants were recruited, with 31 completing the experiments. The remaining 8 participants chose not to continue the experiment due to discomfort from the heat pain. The study included 23 female and 16 male participants, with ages ranging from 22 to 37 years, with an average age of 26.1 (SD 3.57) years. All participants were healthy, and none reported experiencing pain before the experiment.

### Inclusion and Exclusion Criteria

Participants were recruited from the Northeastern University community, including students, faculty, and staff. Inclusion criteria required participants to be between 18 and 50 years of age, in good general health, and not currently experiencing chronic pain or other medical conditions that could interfere with physiological responses. Only English-speaking individuals were included to ensure clear communication and understanding of study procedures. Pregnant individuals were excluded from participation to ensure their comfort and to avoid the introduction of additional physiological variability. There were no exclusion criteria related to gender, race or ethnicity, socioeconomic status, or literacy level.

### Measured Physiological Signals

This study examined two physiological signals, EDA and EMG, to capture responses to pain stimuli.

#### Electrodermal Activity (EDA)

EDA serves as an indicator of neurocognitive stress through changes in the skin’s electrical conductance [28]. Closely linked to the sympathetic branch of the autonomic nervous system, EDA can sense and transmit information about environmental changes, including temperature, pressure, and pain [29-31]. Consequently, EDA reflects emotional and cognitive states, making it a valuable physiological marker across various applications [32].

During emotional arousal or cognitive stress, sweat gland stimulation induces fluctuations in skin conductance, measured by EDA. These changes, largely beyond conscious control, capture subconscious physiological responses to emotions and stress, providing an objective means of assessing an individual’s state [33].

In pain assessment, EDA plays a crucial role by offering a quantitative and objective measure of physiological responses to pain. It provides valuable insights into pain intensity, complementing self-reporting to enhance pain assessment accuracy in research and clinical settings [28,34]. EDA encompasses data related to both slow shifts (tonic component) and the signal’s rapid alterations (phasic changes). Our analysis focused on gathering information from the tonic component, specifically skin conductance level.

#### Electromyography (EMG)

EMG is the electrical signal produced by skeletal muscle activity. These signals originate from motor neurons, which are integral components of the central nervous system. Since EMG signals are a reflection of neuromuscular activity, they find application in the diagnosis of conditions such as muscle injuries, nerve damage, and muscle dysfunction arising from neurological and muscular disorders [35-37]. EMG is an excellent choice for developing an objective pain assessment tool because of its unique ability to measure muscle activity directly. It allows real-time monitoring of muscle responses to understand pain intensity, location, and characteristics [14,38,39].

### Design of the Experiment

The physiological data were collected using the BIOPAC MP160 data acquisition and analysis systems with AcqKnowledge software (BIOPAC Systems, Inc). Smart amplifiers recorded EMG and EDA. Heat stimulation was delivered using OCOOPA Hand Warmers, which offered two temperature settings: 37 and 45 °C. These temperatures were measured and monitored using a BIOPAC SKT (Skin Temperature) Smart Amplifier. Cold stimulation was provided through iced water, with the temperature continuously monitored using a thermometer. In these experiments, heat pain acts as preexisting pain, while cold pain acts as a new pain stimulus.

Using temperature-based modalities for both preexisting (heat) and new (cold) pain stimuli allowed us to design a consistent, safe, and replicable experimental setup. Temperature stimuli are well-established in pain research and offer practical advantages regarding ecological validity and participant safety. Moreover, the thermal approach enabled controlled comparisons of physiological responses across different pain levels while minimizing variability introduced by mechanical or electrical alternatives.

**Figure 1.**
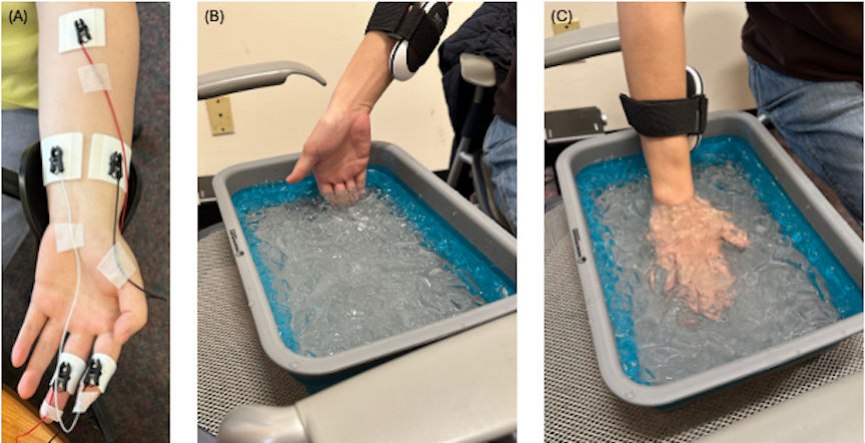
Data acquisition setup and experimental setup for pain stimuli. (A) EDA data were gathered from the ring and index fingers of the participant’s nondominant hand, while EMG data were recorded using three electrodes positioned on the participant’s nondominant forearm. (B,C) Hand warmers, serving as heat pain stimuli, were fastened to the participant’s dominant forearm using a strap. Cold pain stimuli were induced by iced water when participants placed their fingers or hands in the iced water, depending on the stimulus level the participant is expected to receive in the design of experiments: for low-level cold pain stimulus, participants placed fingers in the iced water, and for high-level cold pain stimulus, participants placed the hand in the iced water.

EDA data were collected using the BIOPAC EDA Smart Amplifier attached to the ring and index fingers of the participant’s nondominant hand. Before attaching the sensors to the fingers, the skin was cleaned with wet wipes, and GEL101A was applied to the electrodes to improve conductivity, enhance signal quality, and reduce impedance. EMG data were acquired using the BIOPAC EMG Smart Amplifier, with three electrodes attached to the participant’s nondominant forearm. The skin in the sensor placement area was prepared by cleaning it with wet wipes, followed by abrasion and application of ELPREP. GEL100 was applied to the electrodes to improve contact. To minimize motion artifacts, all cables were secured with medical tape. Hand warmers were fastened to the participant’s dominant forearm using a strap. Figure 1A shows the picture of the placement of the electrodes.

The experiment consisted of two types of pain stimuli: (1) heat pain caused by attaching hand warmers to the forearm and (2) cold pain induced by placing fingers or hands in ice water. Each type of pain had low and high levels. The heat and cold pain stimuli were applied to the dominant hand, while physiological signals were collected from the nondominant hand. At the end of each step, participants were asked to report their pain levels on a scale of 0 to 10. The participants are given a 4-minute relaxation break at the beginning of each data collection session.

We collected baseline data from each participant without inducing any type of pain stimulus. The rest of the experimental procedure consisted of two phases. In the first phase, we collected data from four steps; in Step 1, only the low-level cold pain was applied; in Step 2, only the high-level cold was applied; in Step 3, only low-level heat pain was applied; and in Step 4, only high-level heat pain was applied. In the second phase of the experiments, we applied a different combination of heat and cold pain levels to examine their combined effect in Steps 5 through 8.

The experimental procedures for the first phase involved four steps. First, the participant placed their fingers in iced water and held them there for 8 seconds, representing low-level cold pain. In the second step, they placed their dominant hand in iced water for 8 seconds, representing high-level cold pain. In the third step, using a hand warmer attached to the participant’s dominant forearm, they were subjected to 37 *°*C heat for 1.5 minutes, which caused low-level preexisting heat pain. In the final step of the first phase, using a hand warmer attached to the participant’s dominant forearm, they were subjected to 45 *°*C heat for 1.5 minutes, which caused high-level preexisting heat pain.

The second phase of the experiment involved four additional steps. In the fifth step of the experiment, the participant wore a hand warmer on their nondominant forearm, experiencing a temperature of 37 *°*C for 1.5 minutes. After 80 seconds into the heat pain stimulus, the participant placed their fingers in iced water for 8 seconds. This scenario represents the simultaneous application of low preexisting heat pain and new low cold pain. In the sixth step, the participant repeated Step 5 with the hand warmer on their nondominant forearm, but at a temperature of 45 °C. Again, after 80 seconds, they placed their fingers in iced water for 8 seconds. This scenario represents the simultaneous application of high preexisting heat pain and new low cold pain. In the seventh step, the participant wore the hand warmer on their nondominant forearm at 37 °C for 1.5 minutes. After 80 seconds had elapsed, they immersed their dominant hand in iced water for 8 seconds. This scenario represents the simultaneous application of low preexisting heat pain and new high cold pain. In the eighth and final step, the participant repeated Step 7 with the hand warmer on their nondominant forearm at 45 °C for 1.5 minutes. After 80 seconds, they immersed their dominant hand in iced water for 8 seconds. This scenario represents the simultaneous application of high preexisting heat pain and new high cold pain.

Figure 1B,C illustrates how the hand warmer is positioned on the forearm and how the fingers or hand are placed in the ice water.

### Signal Processing

Both EDA and EMG signals were recorded at a data acquisition rate of 2000 samples per second (2 kHz). For EDA, a low-pass filter with a 1.0 Hz frequency cutoff was used to eliminate high-frequency noise [30,40].

We processed EMG signals through a comb bandstop transformation to eliminate interference from the power line frequency (50 Hz) [41]. The comb bandstop transformation aims to effectively suppress or eliminate interference originating from the power line frequency (50 Hz), ensuring a relatively noise-free EMG signal for analysis and interpretation. Subsequently, a finite impulse response bandpass filter was applied, specifying a low-frequency cutoff at 28 Hz and a high-frequency cutoff at 500 Hz [42]. This step was implemented to filter out both high and low artifacts, such as motion artifacts, and to focus on the EMG signal within the frequency range of 28 to 500 Hz.

Recognizing that the EMG signal centers around 0, a rectified version was generated by averaging samples in sets of 100. This approach makes analysis easy by eliminating negative values and retaining the magnitude of the signal.

To analyze EMG further, the root mean square (RMS) was calculated using a window size of 100 samples. This measurement meaningfully represents the signal’s characteristics because EMG is centered around 0.

### Feature Extraction

In this study, we derived features from EDA and EMG using the “Canonical Time-series Characteristics” outlined by Lubba et al [43]. These features encompass fundamental statistical metrics of time-series data, stationarity measures, entropy, linear correlations, nonlinear time-series analysis techniques, linear and nonlinear model parameters, predictive capabilities, and fits. Specifically, we identified the subset of 22 features highlighted as the most informative by Lubba et al [43]. These features are listed in Table 1. Following all the data processing and extraction steps, we obtained 22 features from EDA, EMG, rectified EMG, and RMS of EMG signals; this resulted in a total of 22×4=88 features. Then, we applied z-transformation to normalize all features for each participant, using the participant-specific mean and SD.

**Table 1.** Time-series feature categories and descriptions using the “Canonical Time-series Characteristics” defined by Lubba et al [43].

Feature category	Features
Distribution	Mode of *z*-scored distribution (5-bin histogram) Mode of *z*-scored distribution (10-bin histogram)
Simple temporal statistics	The longest period of consecutive values above the mean Time intervals between successive extreme events above the mean Time intervals between successive extreme events below the mean
Linear autocorrelation	The first 1/e crossing of the autocorrelation function The first minimum of the autocorrelation function Total power in the lowest fifth of frequencies in the Fourier power spectrum Centroid of the Fourier power spectrum Mean error from a rolling 3-sample mean forecasting
Nonlinear autocorrelation	Time-reversibility statistic, ⟨(*x*_t+1_−*x*_t_)^3^⟩_t_ Auto mutual information, *m=*2*, τ=*5 The first minimum of the auto-mutual information function
Successive differences	Proportion of successive differences exceeding 0.04 *σ* (Mietus et al [44]) The longest period of successive incremental decreases Shannon entropy of two successive letters in equiprobable 3-letter symbolization Change in correlation length after iterative differencing Exponential fit to successive distances in 2D embedding space
Fluctuation analysis	The proportion of slower timescale fluctuations that scale with DFA^a^ (50% sampling) The proportion of slower timescale fluctuations that scale with linearly rescaled range fits
Others	Trace of covariance of the transition matrix between symbols in the 3-letter alphabet Periodicity measure (Wang et al [45])

a DFA: detrended fluctuation analysis.

### Statistical Testing

The initial analysis aims to identify statistically significant features for class differentiation. This includes using the ANOVA test, which assesses variations among the means of various groups. It is applied in various situations to ascertain whether there are any significant differences between the means of the groups [46,47]. The null hypothesis asserts that the means of the groups are the same, while the alternative hypothesis posits that the means are not equal.


(1) $$\begin{array}{rl} H_{0} & :\mu_{1}=\mu_{2}\\ H_{1} & :\mu_{1}\neq \mu_{2} \end{array}$$

We reject the null hypothesis if the calculated *P* value is less than the chosen significance level, say, .05.

We used ANOVA to assess the statistical differences in the means of extracted time series features derived from physiological signals. The sample comprises 31 observations. The normality of data, which is a requisite for ANOVA, is confirmed through the Kolmogorov-Smirnov Test for normality of data and examination of quantile-quantile plots (Q-Q plots) for each individual feature. A significance level of .05 is set for the ANOVA test, which is conducted as a 2-tailed analysis. 

## Results

The following sections present the results of statistical comparisons of EMG and EDA signal features across different combinations of heat and cold pain levels.

### Significant Features in the Presence and Absence of Pre-existing Pain

Table 2 summarizes the statistically significant differences (*P*<.05) in EMG and EDA features across experimental groups. Each row corresponds to a specific hypothesis involving two groups. For example, the first row compares Group 1 (participants who experienced low-level cold pain without preexisting heat pain) with Group 2 (participants who experienced the same low-level cold pain while also experiencing mild preexisting heat pain). This comparison examines feature-level differences across EMG and EDA signals under these two conditions.

**Table 2.** Statistically significant feature categories and the average *P *values of features within each category for different hypotheses, aiming to study the influence of the presence or absence of pre-existing pain on external pain stimuli between symbols in the 3-letter set.

Groups	EMG,^a^ (*P* value)	RMS^b^ of EMG, (*P* value)	Rectified EMG, (*P* value)	EDA,^c^ (*P* value)
Group 1: low-level cold pain without any pre-existing pain Group 2: low-level cold pain with mild pre-existing heat pain	Linear autocorrelation (<.001) Successive differences (.002) Distribution (.02) Others (.006) Statistics (.02)	Linear autocorrelation (.004) Successive differences (.01)	Linear autocorrelation (.003) Successive differences (.001)	Statistics (.02)
Group 1: low-level cold pain without any pre-existing pain Group 2: low-level cold pain with severe pre-existing heat pain	Linear autocorrelation (.004) Successive differences (.005) Others (.02) Statistics (.02)	Linear autocorrelation (.02)	Linear autocorrelation (.02) Successive differences (.03)	Statistics (.02) Others (.04)
Group 1: high-level cold pain without any pre-existing pain Group 2: high-level cold pain with mild pre-existing heat pain	No significant features	No significant features	No significant features	Successive differences (.03)
Group 1: high-level cold pain without any pre-existing pain Group 2: high-level cold pain with severe pre-existing heat pain	No significant features	No significant features	Successive differences (.03)	Others (.03)

a EMG: electromyography.

b RMS: root mean square.

c EDA: electrodermal activity.

For low-level cold pain without any pre-existing pain (Group 1) versus low-level cold pain with mild pre-existing heat pain (Group 2), significant differences were observed in EMG features related to linear autocorrelation, including the “first minimum and the first 1/e crossing of the autocorrelation function.” In the EDA signal, temporal statistics, specifically “time intervals between successive extreme events,” showed statistically significant differences.

For low-level cold pain without any pre-existing pain (Group 1) versus low-level cold pain with severe pre-existing heat pain (Group 2), EMG features related to linear autocorrelation, such as the first minimum and 1/e crossing of the autocorrelation function, were significantly different. The EDA features that showed the differences included “time intervals between successive extreme events” and the “longest period of consecutive values above the mean.”

For high-level cold pain without any pre-existing pain (Group 1) versus high-level cold pain with mild pre-existing heat pain (Group 2), the distinguishing features were found in the EDA signal’s successive differences, particularly the “longest period of successive incremental decreases.”

For high-level cold pain without any pre-existing pain (Group 1) versus high-level cold pain with severe pre-existing heat pain (Group 2), statistically significant differences were observed in the rectified EMG signal for features related to successive differences, including the “change in correlation length after iterative differencing” and the “longest period of successive incremental decreases.” In the EDA signal, differences were observed in the “trace of covariance of the transition matrix between symbols in the 3-letter set.”

### Significant Features in the Mild and Severe Cases of Pre-existing Pain

**Figure 2.**
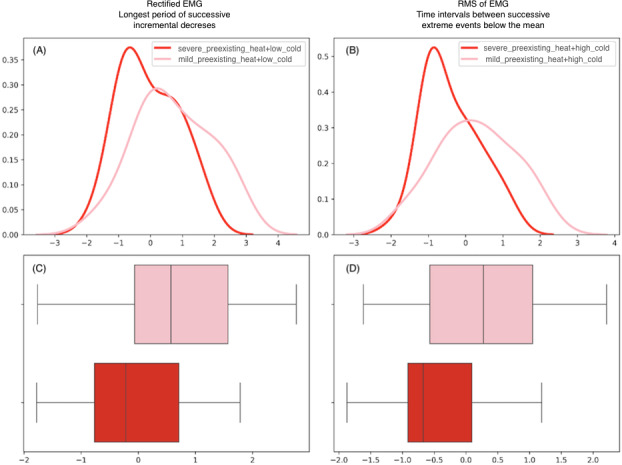
Distribution of features with the influence of pre-existing heat pain: (A,B) Illustrate the probability density of two significant EMG features under low- and high-level cold pain conditions. (C,D) Present the corresponding boxplots for each feature, comparing the mild and severe pre-existing pain conditions. EMG: electromyography; RMS: root mean square.

Table 3 presents the signals and their respective features that exhibit statistically significant differences (*P*<.05) among the groups. In this section, two hypotheses are investigated. The first hypothesis aims to compare physiological signals to assess the influence of mild and severe pre-existing pain in Groups 1 and 2; Group 1 includes signals from participants subjected to low-level cold pain while already experiencing mild pre-existing heat pain; Group 2 includes signals from participants subjected to low-level cold pain while already experiencing severe pre-existing heat pain. The second hypothesis involves comparing the groups to assess the impact of mild and severe pre-existing heat pain on participants when they are subjected to high-level cold pain. Figure 2 visually illustrates the distribution of the most statistically significant features for each of the two hypotheses.

**Table 3.** Statistically significant feature categories and individual features for distinguishing the influence of varying levels of pre-existing pain on the response to low and high levels of cold pain.

Hypotheses and signal		Feature category	Feature (*P *value)
Group 1: low-level cold pain with mild pre-existing heat pain; Group 2: low-level cold pain with severe pre-existing heat pain			
EMG^a^		Distribution	Mode of *z*-scored distribution (10-bin histogram; .03)
RMS^b^ of EMG		Successive differences	Longest period of successive incremental decreases (.01)
RMS of EMG		Statistics	Longest period of consecutive values above the mean (.03)
Group 1: high-level cold pain with mild pre-existing heat pain; Group 2: high-level cold pain with severe pre-existing heat pain			
Rectified EMG		Successive differences	Longest period of successive incremental decreases (.007)
Rectified EMG		Statistics	Longest period of consecutive values above the mean (.01)
EMG		Distribution	Mode of *z*-scored distribution (10-bin histogram; .03)
EMG		Statistics	Time intervals between successive extreme events below the mean (.04)
RMS of EMG		Statistics	Time intervals between successive extreme events below the mean (.005)
RMS of EMG		Statistics	Time intervals between successive extreme events above the mean (.009)
Rectified EMG		Statistics	Time intervals between successive extreme events below the mean (.01)
Rectified EMG		Statistics	Time intervals between successive extreme events above the mean (.01)
Rectified EMG		Successive differences	Change in correlation length after iterative differencing (.03)
EDA^c^		Statistics	Time intervals between successive extreme events below the mean (.01)
EDA		Others	Trace of covariance of transition matrix (.02)

a EMG: electromyography.

b RMS: root mean square.

c EDA: electrodermal activity.

Hypothesis 1 examines the influence of mild and severe pre-existing heat pain on the body’s response to low-level cold pain. Significant differences were observed in the EMG signal’s “mode of *z*-scored distribution.” RMS of EMG showed variations in successive differences and statistics, specifically related to “the longest period of incremental decreases” and “the longest period of consecutive values above the mean.” Similar patterns were found in the rectified EMG signal.

Hypothesis 2 investigates the influence of mild and severe pre-existing heat pain on the body’s response to high-level cold pain. The “mode of *z*-scored distribution” of EMG exhibited significant differences across the groups. RMS of EMG also showed variations in statistics related to “time intervals between successive extreme events below and above the mean.” Rectified EMG signals differed in features pertaining to successive differences and statistics. Additionally, EDA signals showed significant differences in the “trace of covariance of the transition matrix.”

### Heat and Cold Pain Interactions

**Figure 3.**
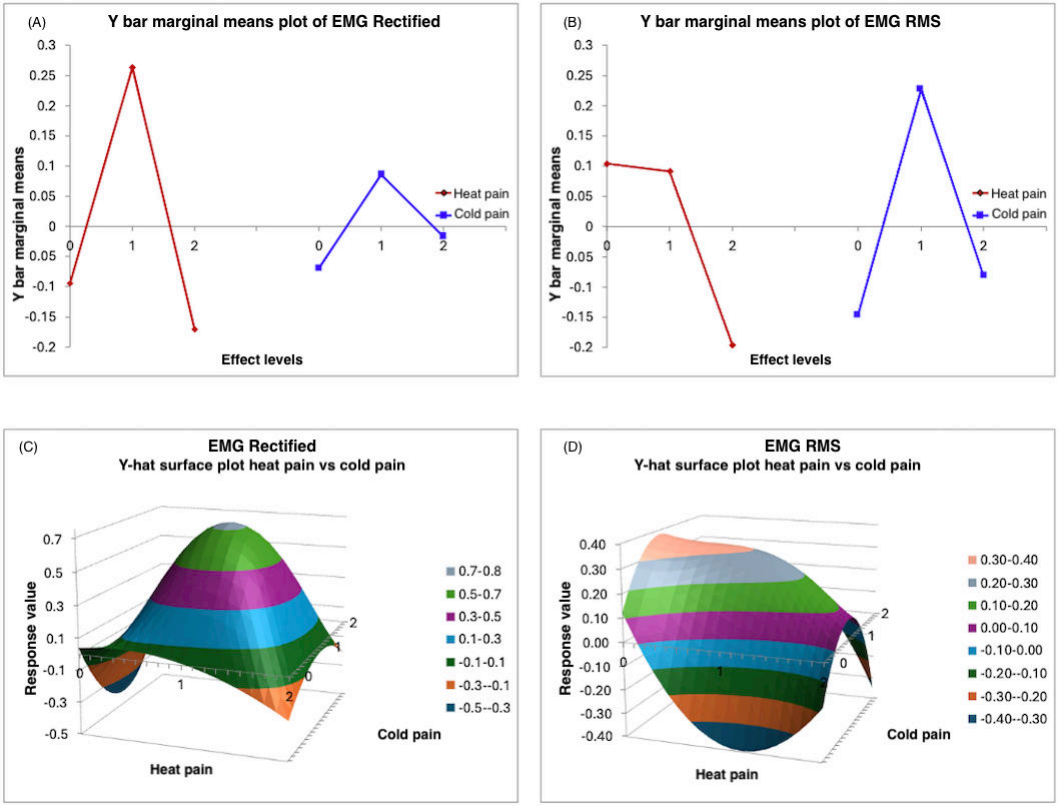
Interaction effects of pre-existing heat pain and new cold pain on EMG features. (A,B) Marginal means plots illustrating how rectified EMG and RMS values vary across different levels of heat pain (0, 1, 2) and cold pain (0, 1, 2). (C,D) Surface plots depicting the variation in responses under various combinations of heat and cold pain levels. EMG: electromyography; RMS: root mean square.

This section presents a response surface analysis using marginal mean plots and surface plots. It examines how varying levels of heat and cold pain affect two statistically significant features: the rectified EMG’s “longest period of successive incremental decreases” and the RMS of EMG’s “time intervals between successive extreme events below the mean.” Figure 3A and C show the rectified EMG response values, while Figure 3B and D display the RMS of EMG response values. The analysis includes pain levels coded as 0 (no pain), 1 (mild pain), and 2 (severe pain).

## Discussion

### Principal Findings

This study was guided by the hypotheses that (1) pre-existing body pain alters the physiological response to a new pain stimulus relative to the physiological response in the absence of pre-existing pain, and (2) pre-existing pain of different intensities produces distinguishably different physiological patterns in response to a new pain stimulus. The use of multimodal physiological signals, EDA, and EMG provides insight into the underlying mechanisms and supports the potential for objective, signal-based pain assessment in complex pain scenarios.

This study found that pre-existing heat pain significantly influences physiological responses to new cold pain stimuli, as indicated by features from EDA and EMG, particularly successive differences, temporal statistics, and distribution features, demonstrating noticeable sensitivity to varying pain combinations.

### Comparative Analysis: Features Sensitive to the Presence and Absence of Pre-existing Pain

This section evaluates how the presence or absence of pre-existing heat pain influences physiological responses when the body encounters a cold pain stimulus. EMG signals exhibit significant variation across groups, particularly in features such as “linear autocorrelation” and “successive differences,” while EDA signals indicate differences through statistical features.

When an external low-level cold pain is applied, the pre-existing heat pain, mild or severe, consistently leads to marked changes in both EMG and EDA signals. In the EMG signal, features such as the “first minimum and the first 1/e crossing of the autocorrelation function” capture distinct temporal characteristics of muscle activity. The first minimum identifies a key point of dissimilarity, while the 1/e crossing reflects the timescale at which the signal’s autocorrelation declines to approximately 36.8% of its peak. In the EDA signal, the “time intervals between successive extreme events” and the “longest period of consecutive values above the mean” emerge as distinguishing features. These results indicate that low-level cold pain elicits prominently different physiological features in the presence and absence of pre-existing heat pain.

When a high-level cold pain stimulus is applied, the EDA signal’s sensitivity to successive differences, particularly the “longest period of successive incremental decreases,” emerges as a distinguishing feature. This feature identifies continuous patterns where EDA consistently decreases from one point to the next and the trace of covariance of the “transition matrix between symbols in the 3-letter set.” This method involves encoding and simplifying the EDA signal into sequences, allowing for the analysis of how these sequences change and relate to each other over time, highlighting its utility in capturing autonomic dynamics influenced by layered pain conditions. Similarly, rectified EMG features tied to successive differences are important: the “change in correlation length after iterative differencing” and the “longest period of successive incremental decreases” further underscore the complementary roles of multimodal physiological measurements.

High-intensity cold pain appears to overshadow the physiological responses associated with pre-existing heat pain. Under these conditions, significant differences are limited and primarily observed in EDA and rectified EMG signals. The overwhelming nature of high-level cold pain reduces the detectability of pre-existing pain effects, making it difficult to distinguish their individual contributions to the physiological response. Despite this, certain features remain sensitive. In the EDA signal, successive differences, particularly the “longest period of successive incremental decreases,” identify continuous patterns where EDA consistently decreases from one point to the next. Additionally, the “trace of covariance of the transition matrix between symbols in the 3-letter” set captures how patterns evolve over time, offering insights into autonomic dynamics under layered pain conditions. Similarly, rectified EMG features related to successive differences, including the “change in correlation length after iterative differencing” and the “longest period of successive decreases,” emphasize the value of combining multimodal physiological measurements to capture subtle effects that may persist despite dominant pain stimuli.

Together, these findings suggest that the influence of pre-existing heat pain on the body’s physiological response is more discernible when cold pain is mild, particularly through EMG and EDA signals. In contrast, high-intensity cold pain may mask these effects, making it difficult to detect the physiological changes due to pre-existing pain. Understanding these interactions between physiological responses due to external and pre-existing pains is essential for interpreting pain states in complex and overlapping pain scenarios. The presence of statistically significant and diverse features supports the notion that pre-existing heat pain has a measurable impact on physiological responses.

### Comparative Analysis: Significant Features in the Mild and Severe Cases of Pre-existing Pain

This section examines how the severity of pre-existing heat pain, ranging from mild to severe, influences the body’s physiological response when exposed to a new cold pain stimulus. The findings reveal distinct alterations in EMG and EDA signals that differentiate these pain intensities.

When participants experience low-level cold pain while the body is already encountering varying degrees of pre-existing heat pain, the physiological responses captured through EMG are particularly sensitive to the severity of pre-existing heat pain. Features like “mode of *z*-score distribution,” which refers to the value or range of values that occur most frequently, indicate shifts in the most dominant muscle activity patterns. Additionally, the RMS of EMG shows differences in features related to successive differences and statistics, specifically the “longest period of incremental decreases” and the “longest period of consecutive values above the mean.” The first feature refers to the duration in the time series where the EMG signal’s RMS consistently decreases incrementally. In simpler terms, it identifies the most extended continuous period during which the RMS values decrease step by step. The second feature pertains to the time series duration in which the EMG signal’s RMS values remain consistently above the mean. This duration captures the longest continuous segment where the RMS values are consistently higher than the average. These features are further supported by similar patterns observed in the rectified EMG signal, reinforcing the robustness of these distinctions.

In the high-level cold pain condition, EMG signals continue to reveal statistically significant differences across pre-existing pain intensities. The “mode of the *z*-scored distribution” emerges as an important marker, indicating distinctive patterns in muscle activity under mild and severe pre-existing heat pain conditions. Analysis of the RMS of EMG signals unveils notable variations in statistics involving “time intervals between successive extreme events above or below the mean.” This observation highlights the complex temporal dynamics associated with the interaction of high-level cold pain and the severity of pre-existing pain. The distribution of rectified EMG signals further reinforces these findings, highlighting distinct patterns in successive differences and statistics, which contribute to the differentiation of the influence of different pre-existing pain conditions. Beyond EMG, EDA signals also contribute to this differentiation. The “trace of the covariance of the transition matrix” emerges as a key feature. This reveals variation in how these patterns evolve over time under different pre-existing pain conditions. The inclusion of EDA signals in our analysis deepens our understanding of physiological responses to the influence of varying pre-existing pain intensities.

The results of this study highlight that the body exhibits distinct responses to cold pain stimuli when experiencing mild versus severe pre-existing heat pain. These findings highlight the intricate relationship between pain conditions and physiological responses. The identified features within EMG and EDA signals offer valuable insights into the body’s mechanisms, highlighting the influence of pre-existing pain on physiological signals.

### Analysis of Heat and Cold Pain Interactions

Response surface analysis provides comprehensive insight into how varying levels of pre-existing heat pain and externally introduced cold pain interact to influence physiological responses.

Figure 3A displays the marginal means plot of the rectified EMG response. Marginal means plots illustrate the responses by considering only the level of one type of pain, independent of the levels of any other type of pain. For example, the response is the strongest when the heat pain is mild. Similarly, when considering only cold pain, the response peaks again at the mildest level of pain. The surface plot represents the interactions between the two types of pain and their effects on the response. The surface plot in Figure 3C reveals a convex shape with a peak, indicating that the rectified EMG responses reach their highest values when both heat and cold pain are at a mild level. The plot shows that the response is low when there is no heat pain and mild cold pain, and similarly low under severe heat pain with no accompanying cold pain.

Figure 3B presents a marginal mean plot of the RMS of EMG responses. Here, it is evident that mild cold levels yield the highest response values. Both “no heat” pain and “mild heat” pain conditions result in high response values, while severe heat pain significantly reduces the RMS of EMG responses. In Figure 3D, surface plots of the RMS of EMG responses are displayed.

Notably, as heat pain increases, the RMS value decreases, reaching its peak when heat pain is absent or mild. Conversely, instances of mild heat paired with no cold pain result in the lowest RMS of EMG response values.

These patterns underscore the importance of considering multidimensional pain contexts, as overlapping pain experiences can interact in nonintuitive ways that meaningfully alter physiological signatures.

### Limitations

The relatively small and homogeneous sample size, consisting of 31 healthy young adults aged 22 to 37 years, is one of the shortcomings of this study. This may limit the generalizability of the findings to broader and clinically relevant populations. Additionally, the study was conducted in a controlled laboratory environment, which may not fully replicate real-world clinical settings, thus limiting its ecological validity. While the use of fixed-intensity heat and cold stimuli was effective for controlled experimental design, it may not capture the full complexity of pre-existing pain conditions and individual pain thresholds. Furthermore, the fixed-intensity nature of these stimuli does not account for interindividual variability in pain sensitivity, which could influence physiological responses. The devices used in this study did not support personalized stimulus calibration, which we recognize as a limitation.

### Future Directions

Future work is open to expanding the sample population to include individuals from diverse age groups and clinical backgrounds, particularly those experiencing chronic or postsurgical pain, to improve the generalizability of findings. Validation in real-world clinical environments is also crucial for enhancing ecological validity. To better reflect the complexity of pain experiences, future studies should explore alternative or multimodal pain induction methods beyond heat and cold stimuli and incorporate personalized calibration to account for individual pain thresholds. Additionally, expanding the range of physiological signals—such as heart rate variability, electroencephalography, and functional neuroimaging—may offer a more comprehensive understanding of the neural and autonomic correlates of pain.

This study used statistical analysis to examine the significance of physiological differences across pain conditions. In future work, we will further explore machine learning models to analyze physiological responses to new external pain stimuli. This approach will enable us to assess the intensity of pre-existing pain caused by chronic conditions, injuries, or surgeries. By integrating machine learning, we aim to develop predictive models that can objectively assess pain intensity and support personalized, effective pain management, particularly in clinical settings where patients are unable to verbally communicate their pain levels.

### Conclusions

Accurate pain assessment is crucial for the correct diagnosis and effective treatment of many diseases. While existing literature has developed tools for estimating pain levels based on physiological responses, these studies often focus on healthy individuals experiencing acute pain, overlooking the potential influence of pre-existing conditions, such as postsurgical pain, chronic pain, and physical discomfort, on the physiological signals triggered by acute pain. Acknowledging this factor is essential, as individuals may respond differently to new pain stimuli depending on the intensity of their pre-existing pain.

This study examined the impact of pre-existing heat pain through experimental research when participants were exposed to cold pain stimuli. We used heat pain as the pre-existing pain condition, cold pain as the new pain stimulus, and EMG and EDA as physiological signals. By using statistical tests, we observed significant differences in specific EDA and EMG signal features across varying levels of pre-existing heat pain and new cold pain combinations. Notably, simple temporal statistics (the most extended period of consecutive values, time intervals between successive extreme events), successive differences (change in correlation length after iterative differencing), distribution (mode of *z*-scored distribution), and autocorrelation (the first 1/e crossing of the autocorrelation function) emerged as primary feature categories that significantly varied across pre-existing heat pain and new cold pain intensity combinations.

Our investigation into the differences in EMG and EDA signals in the presence of different levels of pre-existing heat pain has revealed valuable insights. The distinction between the absence of pre-existing pain and the presence of mild or severe pre-existing heat pain, particularly when stimulated with new low-level cold pain, highlighted statistically significant differences in both EMG and EDA signals. Notably, when we switched to high-level cold pain, EDA emerged as a more reliable indicator of variation in pre-existing pain than EMG. During high-level cold pain, the time series features of “successive differences” proved to be effective indicators of the level of the pre-existing pain. Furthermore, our analysis of mild and severe pre-existing heat pain scenarios revealed that EMG exhibited statistically significant differences, particularly in response to the new low-level cold pain, whereas EDA remained relatively unchanged. However, when we switched to high-level cold pain, both EMG and EDA signal features exhibited statistically significant differences. Successive difference, temporal statistics, and distribution features of time series emerged as reliable indicators of the pre-existing heat pain in these cases. These findings shed light on the changes in EMG and EDA signals across different levels of pre-existing pain, advancing our understanding of physiological responses in pain assessment.

## Abbreviations

EDA: electrodermal activity
EMG: electromyography
RMS: root mean square
